# Investigating links between creativity anxiety, creative performance, and state-level anxiety and effort during creative thinking

**DOI:** 10.1038/s41598-023-39188-1

**Published:** 2023-10-10

**Authors:** Richard J. Daker, Indre V. Viskontas, Grace F. Porter, Griffin A. Colaizzi, Ian M. Lyons, Adam E. Green

**Affiliations:** 1 Department of Psychology, Georgetown University, Washington, D.C., USA; 2 Department of Psychology, University of San Francisco, San Francisco, USA; 3 Department of Psychology, Northeastern University, Boston, USA

**Keywords:** Psychology, Human behaviour

## Abstract

Identifying ways to enable people to reach their creative potential is a core goal of creativity research with implications for education and professional attainment. Recently, we identified a potential barrier to creative achievement: creativity anxiety (i.e., anxiety specific to creative thinking). Initial work found that creativity anxiety is associated with fewer real-world creative achievements. However, the more proximal impacts of creativity anxiety remain unexplored. In particular, understanding how to overcome creativity anxiety requires understanding how creativity anxiety may or may not impact creative cognitive performance, and how it may relate to state-level anxiety and effort while completing creative tasks. The present study sought to address this gap by measuring creativity anxiety alongside several measures of creative performance, while concurrently surveying state-level anxiety and effort. Results indicated that creativity anxiety was, indeed, predictive of poor creative performance, but only on some of the tasks included. We also found that creativity anxiety predicted both state anxiety and effort during creative performance. Interestingly, state anxiety and effort did not explain the associations between creativity anxiety and creative performance. Together, this work suggests that creativity anxiety can often be overcome in the performance of creative tasks, but likewise points to increased state anxiety and effort as factors that may make creative performance and achievement fragile in more demanding real-world contexts.

## Introduction

Creative ability is one of the most sought-after qualities among employers across industries^1^, and for good reason. The ability to think creatively—often defined as thought that results in the generation of novel and useful products^2^—drives innovation at individual, organizational, and societal levels. Research that allows for the identification, and eventually the mitigation, of potential barriers to optimal creative thinking—allowing people to reach their creative potential—should therefore be prioritized.

One potential barrier to the fulfillment of creative potential, which we have recently identified, is “creativity anxiety”, or anxiety about thinking creatively^3^. We developed and validated the Creativity Anxiety Scale (CAS), and found evidence that creativity anxiety can manifest across a variety of domains, from traditionally “creative” domains like music and art to domains that are often considered more technical than creative, like science and math. Both in general and across a variety of domains, we also found evidence that situations that involve a need to think creatively elicited higher anxiety ratings than similar situations that did not necessitate creative thinking. This finding suggests that adding a need to be creative to a task is, on average, anxiety-inducing. Perhaps most concerning, we found evidence that creativity anxiety was associated with a tendency to report lower levels of real-world creative achievement^3,4^, as measured using the Creative Achievement Questionnaire^5^. This work provided preliminary evidence that creativity anxiety may very well act as a barrier to creative thinking.

While this previous work supplied important evidence that creativity anxiety was predictive of creative *achievement* (i.e., a combination of engagement with and success in creative pursuits), no work has yet investigated the relationship between creativity anxiety and performance on measures of creative *ability* (i.e., measures that test one’s ability to think creatively). While it may seem as if creative achievement and creative ability would go hand-in-hand, past work has found that many measures of creative thinking ability are often uncorrelated with measures of creative achievement^6^. This suggests that links between creativity anxiety and creative achievement and ability should be treated as two separate lines of inquiry—the former focused on real-world degrees of creative accomplishment and the latter focused on the cognitive consequences creativity anxiety can have on in-the-moment creative thinking. There is substantial reason to think that those who are high in creativity anxiety may display worse creative performance. First, drawing on a corresponding line of research on anxiety specific to math (i.e., math anxiety), there is a reliable association between math anxiety and math performance^7–9^. The same is true of other “cognition-specific anxieties” as well—spatial anxiety is negatively predictive of spatial reasoning performance^10,11^, in children reading anxiety is negatively predictive of reading performance^12^, foreign language anxiety is negatively predictive of foreign language performance^13^, etc. Assuming that creativity anxiety functions similarly to these other cognition-specific anxieties, creativity anxiety should therefore negatively predict creative performance. Multiple explanations have been proposed to explain these associations. Some have focused on avoidance—those who are anxious about a type of thinking tend to avoid situations that involve that type of thinking and therefore receive less practice with it ^14,15^. Other accounts are focused on state-level impacts—feeling anxious about a type of thinking might mean higher state anxiety levels when faced with a situation that involves that type of thinking^13–15^ (see below for additional detail). Second, meta-analytic work has shown that general anxiety is negatively predictive of creative performance^16^. Again, multiple explanations for this have been posited. Some theories focus on working memory deficits caused by anxiety^17^ while others hold that anxiety is linked to poor creativity as a result of behavioral inhibition^18^. Given that other work has found that cognition-specific anxieties are more strongly predictive of cognitive performance than is general anxiety^10^, it thus is reasonable to predict that creativity anxiety would be associated with creative performance. The chief goal of the present work was to assess the extent to which creativity anxiety is, indeed, predictive of worse creative performance. We were also interested in exploring whether an increase in state anxiety or a decrease in effort, or a combination of both, might serve as a mechanism for how creativity anxiety affects performance.

To that end, another goal of the present work was to begin to understand the state-level experience of creative performance for those high in creativity anxiety. Drawing again from the math anxiety literature, one of the chief mechanisms that has been proposed to explain why math-anxious people underperform in math is increased state anxiety^19–21^. By this account, when faced with math, those who are math-anxious experience an increase in state anxiety, and this state anxiety in turn interferes with cognitive processing that is necessary to do cognitively-demanding math. A similar dynamic may be at play when someone who is high in creativity anxiety is faced with a need to be creative. However, no prior work has investigated whether those who are higher in trait-level creativity anxiety experience heightened state anxiety during creative performance. This is an important gap in the literature to fill, as it is a key assumption of the Creativity Anxiety Scale that those who indicate they would be anxious in hypothetical situations that involve creative thinking actually do become more anxious in-the-moment when they have to think creatively^3^. As a result, this investigation enables us to perform an additional test of the validity of the Creativity Anxiety Scale. If it were the case that those higher in creativity anxiety experienced heightened state anxiety while completing creative tasks, this could mean their creative performance may be particularly fragile in cognitively demanding contexts.

In addition to understanding whether the degree of in-the-moment anxiety during creative performance is linked to creativity anxiety levels, we also wished to investigate whether creativity anxiety is associated with the degree of self-reported effort associated with creative performance. There is evidence in the math anxiety literature, for instance, that those who are math-anxious may be less likely to expend effort on tasks that involve math^22,23^. This avoidance is thought to occur as a form of emotion regulation—if you are anxious about something, expending less effort on it in situations where you cannot avoid it entirely can be a way to reduce how anxiety-inducing that thing is^23^. If this were the case for creativity anxiety, this could be another mechanism by which creativity anxiety might be linked to creative performance. In contrast, there is evidence in the literature on general anxiety that those who are highly anxious engage in *additional* effort when completing cognitive tasks as a way to compensate for the otherwise negative effects of anxiety on performance^24^. Here, the idea is that anxiety can be a motivating factor. Beginning to understand how the state-level experience of creative performance might differ as a function of creativity anxiety can provide useful mechanistic insights that could inform future intervention work. In one study described here, we therefore tested whether A) creativity anxiety was predictive of state anxiety and/or effort associated with creative tasks and B) if so, whether these associations could explain any observed links between creativity anxiety and creative performance. Understanding the state-level impacts of creativity anxiety can provide important mechanistic insight that can inform interventions that aim to reduce its harmful effects on creative achievement.

To test whether creativity anxiety is, indeed, linked to poor creative performance, we measured creativity anxiety alongside performance on three different creativity tasks. We included multiple tasks for a few reasons. First, no one task will adequately capture all of “creative performance” on its own. A small battery of tasks is not fully adequate to capture such a complex construct, but the inclusion of multiple tasks that measure different facets of creativity will allow for a fuller picture than using one task alone. Second, there may very well be differences in the extent to which creativity anxiety is associated with performance on different creative tasks. For instance, past meta-analytic work has found significant differences in the strength of the association between general anxiety and different creativity tasks with tasks that were coded as more complex (and therefore more cognitively demanding) having stronger negative associations^16^. By including multiple measures of creative performance, we can test whether creativity anxiety is differentially predictive of performance on tasks that measure different facets of creativity.

One task we included in our battery was the Alternative Uses Task (AUT^25^), which is a standard task in the creativity literature used to measure primarily divergent creative thinking. In this task, participants are given a common household object and asked to generate as many alternative uses for this object as possible in a given timeframe, and participants are scored on both the number of different ideas they generate (fluency) and how original those ideas are (originality). Our battery also included items from the Compound Remote Associates task (CRA^26^), another standard task in the creativity literature that is often used to measure primarily convergent creative thinking. The CRA is modeled after Mednick’s Remote Associates Test (RAT^27^). In the CRA, participants are shown three words (ex. “pine, crab, sauce”) and asked to generate a fourth word that, when combined with each of the three stimulus words, produces a compound word or phrase (ex. “apple”; “pine-apple”, “crab-apple”, “apple-sauce”). This task differs from the AUT in that, whereas there are no right or wrong answers on the AUT, on the CRA there is a single correct answer, and participants must think creatively to arrive at that answer.

We further tested creative performance on the Analogy Finding Matrix task developed by Weinberger et al^28^. In this task, participants are presented with a large matrix of possible analogies (ex. “Kitten is to Cat as Puppy is to Dog”) and asked to select the valid analogies (including this example). In each matrix, there are 100 possible analogies, but only 17 of them are valid. Crucially, the valid analogies differ from each other in how “creative” they are, as quantified via semantic distance^29,30^. “Kitten is to Cat as Puppy is to Dog” is a less creative (less semantically distant) analogy, whereas “Kitten is to Cat as Spark is to Fire” is a more creative analogy. To do well on this task, participants must therefore be able to think creatively as they construct their analogies. Participants completed iterations of the Analogy Finding Matrix task under two conditions: uncued (no instruction regarding creativity), and creativity-cued (participants were prompted to “think creatively” as they constructed their analogies). Creativity cueing has been found to boost performance on this task^28,31^. One interesting question that can be investigated by including both a creativity-cued and uncued version of this task is whether those who are high in creativity anxiety are less able to consciously augment their creative state when prompted to be creative. Additionally, performance on this task can be thought of as including a more balanced mix of divergent and convergent reasoning compared to the AUT and CRA, which place their focus more on one type of reasoning over the other (though neither are process-pure measures of “divergence” or “convergence”^32^). Finally, performance on the uncued task (in which participants are not explicitly told to be creative) can act as a measure of how creatively participants think when not prompted to be creative. It is possible that those who are high in creativity anxiety are less likely to spontaneously think creatively in situations that allow for, but do not explicitly demand, creative thinking.

We collected these measures from three different samples of university students. In our third sample, we went beyond simply measuring creativity anxiety levels alongside creative performance measures and additionally collected information about state-level factors that might be relevant for performance: state anxiety and effort. After each task, we asked participants to report both how anxious they felt while they were completing the task and how much effort they put into completing the task. This protocol allowed us to not only investigate associations between creativity anxiety and creative performance, but also between creativity anxiety and state-level dynamics that may be consequential for creative performance. Moreover, collecting these measures together allowed us to test whether any observed associations between creativity anxiety and creative performance that we did see could be attributed to either state-level anxiety or effort as mechanisms. Understanding whether creativity anxiety is associated with poor performance in specific types of creative thinking—and gaining an initial understanding of state-level anxiety and effort dynamics of creative performance for those higher in creativity anxiety—is the first step toward the development of effective interventions to overcome this barrier and allow people to perform to the best of their creative ability.

## Methods

### Participants

Due to the COVID-19 pandemic, all data collection took place online, and past work has found that effect sizes of online studies tend to be smaller than similar in-lab studies^33^. To compensate for this trend, a total of 506 participants were recruited in three separate studies; this large sample size allowed us to detect correlations as small as *r* = .124 with .8 power at an alpha of .05. In Study 1, 153 participants (71 female, mean age = 20.57, SD = 1.47) were recruited from computer science courses at Georgetown University as part of a broader study aimed to assess predictive effects of creative abilities and attitudes on performance in computer science courses (for details, see^34^. In Study 2, 216 participants (173 female, mean age = 20.19, SD = 2.84) were recruited from the Georgetown University Psychology Department participant pool. In Study 3137 participants (104 female, mean age = 20.01, SD = 1.11) were recruited as part of the University of San Francisco’s Psychology Department participant pool. All students were compensated with extra credit in their academic courses for participating. All participants provided informed written consent to participate. All procedures for research carried out at Georgetown University were approved by Georgetown University’s Institutional Review Board, and all procedures for research carried out at the University of San Francisco were approved by the University of San Francisco’s Institutional Review Board. All research was performed in accordance with the relevant guidelines and regulations.

### Procedure

Participants completed a battery of questionnaires and cognitive tasks online. All questionnaires and cognitive tasks were presented in a randomized order. The total battery took, on average, 35 min to complete, and participants were instructed that they could take breaks in between tasks or questionnaires but that they should refrain from taking breaks while in the middle of a measure.

### Measures

#### Survey measures

##### Creativity anxiety scale

Participants completed the Creativity Anxiety Scale (CAS^3^). The CAS is comprised of two item types: creativity anxiety items (CA) and non-creativity anxiety control items (NAC). On each item, participants are presented with a situation and asked to rate how anxious this situation would make them feel on a scale from 0 (None at all) to 4 (Very much). Each of the 8 CA items measure anxiety toward situations that require being creative (for example “Having to come up with a unique way of doing something” and “Having to think in an open-ended and creative way”). Each of the 8 NAC items measure anxiety toward similar situations as those presented in the CA items but that remove the need to be creative (for example, “Having to precisely follow an established method of doing something” and “Having to think in a precise and methodical way”). The inclusion of the NAC items allows for anxiety toward the noncreative demands of the situations presented in the CA items to be measured and controlled for. Both CA and NAC scores range from 0 to 32, where higher scores indicate greater anxiety. Cronbach’s α was .90 for both the CA and NAC measures.

##### Trait component of the state-trait anxiety inventory

Participants completed the trait component of the State-Trait Anxiety Inventory^35^. Participants were presented with a number of statements intended to probe the anxiety-related feelings they generally experience (for example, “I feel that difficulties are piling up so that I cannot overcome them” and “I feel pleasant” [reverse scored]) and asked to indicate how often they feel what is described in each statement on a scale from 1 (Almost never) to 4 (Almost always). This 20-item scale ranges in scores from 20 to 80, where higher scores indicate greater anxiety. This scale was included for use as a covariate when assessing associations between creativity anxiety and other measures to ensure that any associations are not driven by shared associations with general trait anxiety. Cronbach’s α was .93 for this measure.

#### Cognitive Performance Measures

##### Alternative Uses Task

Participants’ ability to generate unique ideas was measured using the Alternative Uses Task developed by Guilford (AUT^25^). Participants were given the following instructions: “For this section, list as many alternative uses for a brick as you can think of in two minutes.” Participants typed their responses. We extracted two dependent variables from the AUT: Fluency, or the number of unique responses participants generated, and Originality, or how original, on average, those responses were. Both of these scores were determined by trained independent raters. In Study 1, two independent raters provided ratings, and for Studies 2 and 3, three independent raters provided ratings. For each response a participant gave, each independent rater provided an Originality rating (possible scores ranged from 1 “very obvious and ordinary use” to 5 “very imaginative, re-contextualized use”) and a Fluency rating (0 if the response was inappropriate or irrelevant, 1 if the response was appropriate and relevant). Inter-rater reliability was moderate or high for each dependent variable in each study (Intra-class correlation coefficients: Study 1—Originality = .83, Fluency = .99; Study 2—Originality = .87, Fluency = .99; Study 3—Originality = .77, Fluency = .97). To be considered a valid response, at least half of the raters must have indicated a 1 for Fluency. An overall Fluency score was obtained for each participant by summing the amount of valid responses that participant produced. Originality scores for each participant were generated by taking the average Originality ratings of all valid responses.

##### Compound remote associates

Participants completed the items from the Compound Remote Associates task (CRA^26^), which was modeled after the Remote Associates Test (RAT^27^). On each trial, participants were given three stimulus words and are told to generate a fourth word that would create a common phrase when combined with each of the stimulus words. For example, participants were presented with a word triad such as [opera/hand/dish] and generated a fourth word, in this case, [soap], which makes three possible compound words/phrases with the original triad: soap opera, hand soap, and dish soap. Participants were given 10 s for each trial to type their response. Participants completed a total of 15 trials that had an average solution rate of 49.2% (with a range of solution rates from 29 to 79%) in a norming study^26^. In the present work, we observed an average solution rate of 41%. Fifteen participants either did not make any responses during this task or did not complete the task according to the instructions (e.g., several participants provided a series of three words in response to each trial when the task was to generate a single word that linked the three words shown as the stimulus) and were therefore excluded from analysis involving the CRA.

##### Analogy finding matrix task

Creative analogical reasoning was tested using the Analogy Finding Matrix task^28,31^. In this task, participants are shown a matrix of word-pairs, with 5 word-pairs shown as a column on the left side of the screen (stem pairs) and 20 word-pairs shown as a row at the top of the screen (completion pairs). Participants received the following instructions: “Your task is to make analogies by combining word-pairs on the left side of the grid with word-pairs along the top of the grid. Each word-pair should be read as ‘[Top Word] is to [Bottom Word]’. For example, ‘Helmet is to Head.’ Check the boxes to indicate when a word-pair from the top combines with a word-pair on the left to make a valid analogy. Try to make as many analogies as you can. However, only valid analogies should be listed, so don’t list analogies unless you can describe how the two word pairs are analogous.” Each stem pair in this task can be combined with 3 or 4 of the completion pairs to form valid analogies. A total of 17 valid analogies can be found in each matrix (out of 100 possible combinations of stem pairs and completion pairs; 83 possible invalid combinations). The valid analogies range in the semantic distance between the two analogs (word-pairs). Semantic distance is commonly used as a quantitative index of creativity, based on the similarity or distinctness of the context of word usage across corpora of text representing mass aggregations of a written language^36,37^ (in this case, English). For example, the valid analogy “[Kitten] is to [Cat] as [Puppy] is to [Dog]” is low in semantic distance and is therefore considered a relatively uncreative analogy while the valid analogy “[Kitten] is to [Cat] as [Spark] is to [Fire]” is high in semantic distance and is therefore considered a relatively creative analogy.

After completing one analogy matrix with the above instructions, participants in the Analogy Finding Matrix task are presented with another analogy matrix, this time with the following additional instructions: “This time, please think creatively as you search for valid analogies. Some analogies may not be obvious right away, so be sure to look for abstract connections.” Previous work has shown that this “creativity cue” increases the number of valid analogies participants construct without significantly increasing the number of invalid analogies that are constructed^28,31^. Two versions of the Analogy Finding Matrices were used, and the order in which they were presented was counterbalanced. Although the matrix versions were counterbalanced, the cued condition always came second. Note that as a component of the study by Weinberger et al.^28^, both matrix versions were presented without the creativity cue and no evidence of practice effects was found, suggesting that differences in performance on the cued and uncued matrices can be attributed to the presence of the creativity cue rather than to practice. Participants were given 3 min for each Analogy Finding Matrix version.

Three separate dependent variables were produced by this task: the number of correct analogies selected on the uncued matrix (Analogy Finding Matrix—Uncued), which provides a measure of creative analogical reasoning ability when not explicitly prompted to be creative; the number of correct analogies selected on the matrix with the creativity cue (Analogy Finding Matrix—Creativity Cue), which provides a measure of creative analogical reasoning ability when explicitly prompted to be creative; and the difference between these scores (Analogy Finding Matrix—Cue Effect), which provides a measure of how much participants were able to increase their creative analogical reasoning ability after receiving a prompt to be creative. When assessing associations between these variables and creativity anxiety, we residualized out the number of invalid analogies participants constructed to penalize for incorrect responding. This was done because otherwise a hypothetical participant who selected a large number of options at random could score highly on this task despite not actually engaging in any analogical reasoning. Six participants did not select any analogies on at least one version of this task and were therefore excluded from analyses involving the Analogy Finding Matrix task.

##### Raven’s advanced progressive matrices

Participants completed a portion of the Raven’s Advanced Progressive Matrices (Raven’s^38^). This task requires participants to complete a visual pattern in which one section of nine is missing by selecting one of eight options to fill in the missing section. One half of the Raven’s questions (all even numbered questions) were selected for this for participants to complete. After completing a practice problem, participants were given 10 min to complete as many of the Raven’s items as possible (17 total). Scores were calculated by summing the total correct responses. This task was included for use as a covariate measure of fluid intelligence when investigating links between creativity anxiety and measures of creative performance. Two participants did not make any responses during this task and were therefore dropped from analyses involving Raven’s.

#### State-level measures of anxiety and subjective effort

In Study 3, after each task, participants were asked to indicate how anxious they were during the task and how much effort they expended during the task. The exact questions were: “While you were completing the previous task, how much anxiety did you feel?” (anxiety) and “While you were completing the previous task, how much effort did you put in?” (effort). Response options to both questions were as follows: “None at all” (0), “A little” (1), “A fair amount” (2), “Much” (3), “Very much” (4). Past work has used similar measures of state anxiety^39^ and subjective effort^40^. Inclusion of these measures allowed us to assess associations between creativity anxiety and state-level factors associated with performing creative tasks. One participant did not respond to the state-level prompts and was therefore excluded from analyses involving these measures.

## Results

### Analysis 1: Testing associations between creativity anxiety and measures of creative performance

For descriptive statistics of all measures, see Table 1. The goal of Analysis 1 was to test the extent to which creativity anxiety was associated with multiple measures of creative performance. Our approach was to assess zero-order associations between creativity anxiety and each measure of creative performance, as well as assessing partial correlations controlling for non-creativity anxiety control scores, general trait anxiety, performance on Raven’s Progressive Matrices, and dummy variables indicating the studies from which participants were aggregated.

**Table 1:** Descriptive statistics for all variables are shown. *This measure reflects the difference in performance between the uncued and creativity-cued analogy finding matrix tasks.

Measure	N	Mean (SE)	Skew	Kurtosis
Creativity anxiety	506	13.15 (.28)	.18	.36
Non-creativity anxiety control	506	9.04 (.28)	.70	.21
General trait anxiety	506	26.34 (.44)	.05	− .34
AUT fluency	506	8.72 (.18)	.95	3.23
AUT originality	506	2.13 (.02)	− .19	.80
CRA	491	5.97 (.15)	− .06	− .83
Analogy finding matrix—no creativity cue	500	8.09 (.17)	.31	− .92
Analogy finding matrix—creativity cue	500	9.13 (.18)	− .11	− .97
Analogy finding matrix—creativity cue effect*	500	1.04 (.13)	.18	.36
Raven’s	504	9.33 (.14)	− .27	− .33

**Figure 1:**
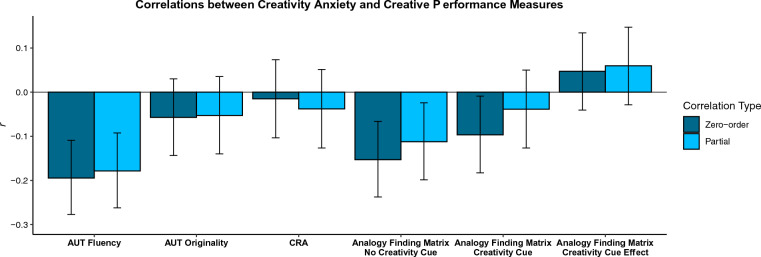
Shows associations between creativity anxiety and measures of creative performance. The darker blue bars indicate zero-order Pearson’s correlations. The lighter blue bars indicate partial correlations controlling for the following covariates: non-creativity anxiety control scores, general trait anxiety, Ravens performance, and dummy variables indicating which of the three studies participants were from. Error bars represent 95% confidence intervals.

Results (Fig. 1) indicated that creativity anxiety was significantly associated with both AUT Fluency (*r*(504) = − .195, *p* = 1E−5; *r*_*partial*_(497) = − .179, *p* = 6E−5) and with Analogy Finding—No Creativity Cue (*r*(498) = − .153, *p* = 6E−4; *r*_*partial*_(491) = − .112, *p* = .013). There was also a significant zero-order association between creativity anxiety and Analogy Finding—Creativity Cue (*r*(498) = − .097, *p* = .031), but this association did not hold after controlling for covariates (*r*_*partial*_(491) = − .039, *p* = .391). No other significant associations between creativity anxiety and measures of creative performance were observed (all *p*s >  .05; note also that there was no association between creativity anxiety and Raven’s performance).

These results suggest that creativity anxiety is negatively associated with the number of unique ideas individuals can generate (indexed by AUT Fluency), but not with how creative those ideas are (indexed by AUT Originality). They also suggest that those who are higher in creativity anxiety are less likely to spontaneously make creative connections between distant concepts if not prompted to be creative (indexed by Analogy Finding Matrix—No Creativity Cue), potentially reflecting lower baseline levels of “state creativity”^41^. If prompted to be creative, however, those higher in creativity anxiety appear to be just as able to make creative connections between concepts as those lower in creativity anxiety (indexed by Analogy Finding Matrix—Creativity Cue), and they also appear just as able to augment their creative state (indexed by the Analogy Finding Matrix—Creativity Cue Effect). Those higher in creativity anxiety also appear to be just as skilled at engaging in primarily convergent creative reasoning (indexed by CRA performance) as those who are less anxious about thinking creatively.

Together, these results suggest that the links between creativity anxiety and creative performance are complex: there are some modest negative associations, but it is certainly not the case that those who are anxious about thinking creatively underperform on all tasks involving creative thinking. The lack of negative associations between creativity anxiety and certain forms of creative performance raises additional questions: why is it that those who are anxious about thinking creatively would perform just as well as those who are not anxious?

One possibility is that those who were high in creativity anxiety simply did not become anxious in the moment when completing all of these creativity tasks. It is possible, for instance, that participants who are high in creativity anxiety experienced heightened state anxiety in response to the AUT, but not in response to the CRA, possibly due to differing perceptions of how much each task actually involved “creative thinking”. Another possibility could be that those who are anxious about creative thinking engage in compensatory effort to make up for their anxiety, which has been found to occur in the case of general anxiety in past research^24^. Contrastingly, there is also evidence in the math anxiety literature that those who are math-anxious are more likely to expend little effort during math performance as a form of “micro-avoidance” of math, the target of their anxiety^22,42^. It may therefore be the case that those who are high in creativity anxiety are less likely to expend effort during creative performance.

Addressing these questions requires getting state-level information about participants’ anxiety and effort levels associated with each task. Thus, in Study 3, we included state-level measures of anxiety and effort in which we asked participants about their experience of each task directly after the task ended. This protocol afforded the opportunity not only to test why we may not have seen predicted associations between creativity anxiety and certain types of creative performance (paths from creativity anxiety to state-level factors to creative performance may not be intact), but also to test whether state anxiety or effort can explain any of the associations we did find (e.g., Does heightened state anxiety explain the association between creativity anxiety and AUT Fluency?). In the next section, we make use of these state-level measures to better understand the observed associations (or lack thereof) between creativity anxiety and measures of creative performance.

### Analysis 2: State-level correlates of creativity anxiety and creative performance

The goal of this analysis was to assess correlations between creativity anxiety and both state anxiety levels and effort levels participants reported during each of the creativity tasks. We also planned to test whether state anxiety and/or effort could explain associations (or lack of associations) between creativity anxiety and creative performance. Of the three studies included as part of the present work, only Study 3 included state-level measures. For descriptive statistics of all state-level measures, see Table 2. Note that there was no evidence of ceiling or floor effects for any measures.

**Table 2:** Descriptive statistics for all state-level variables are shown. *These measures reflect the difference in ratings between the uncued and creativity-cued Analogy Finding Matrix tasks.

Measure	N	Mean (SE)	Skew	Kurtosis
AUT state anxiety	136	1.68 (.10)	.38	− .80
CRA state anxiety	136	2.24 (.11)	− .03	− 1.23
Analogy finding matrix—no creativity cue state anxiety	136	1.32 (.10)	.69	− .53
Analogy finding matrix—creativity cue state anxiety	136	1.07 (.09)	.75	− .01
Analogy finding matrix—creativity cue effect state anxiety*	136	− .25 (.09)	− .33	.33
AUT effort	136	2.56 (.08)	− .14	− .52
CRA effort	136	2.78 (.09)	− .48	− .28
Analogy finding matrix—no creativity cue effort	136	2.29 (.09)	− .18	− .40
Analogy finding matrix—creativity cue effort	136	2.46 (.09)	− .28	− .46
Analogy finding matrix—creativity cue effect effort*	136	.16 (.07)	.21	1.86

**Figure 2:**
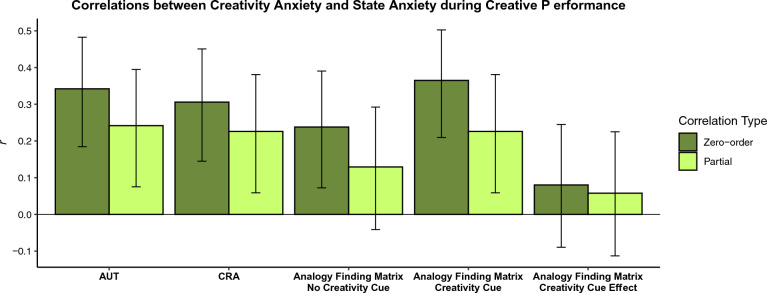
Shows associations between creativity anxiety and levels of state anxiety participants reported experiencing during creative performance. The darker green bars indicate zero-order Pearson’s correlations. The lighter green bars indicate partial correlations controlling for non-creativity anxiety control scores and general trait anxiety. Error bars represent 95% confidence intervals.

First considering associations between creativity anxiety and state anxiety during creative performance, Fig. 2 shows both zero-order correlations between creativity anxiety and state anxiety during each task and partial correlations controlling for non-creativity anxiety control scores and for general trait anxiety. Results indicated significant zero-order associations between creativity anxiety and all state anxiety measures (all *p*s < .05). Moreover, these associations remained significant after controlling for non-creativity anxiety control scores and general trait anxiety for all measures except for Analogy Finding Matrix—No Creativity Cue (this lack of association could be because participants were less likely to perceive the Analogy Finding Matrix task as “creative” when it is not accompanied by a creativity cue). Together, these results provide important evidence that creativity anxiety is, as expected, associated with state anxiety during creative tasks—even including tasks for which there was not a significant association between creativity anxiety and performance, like the CRA. We did not observe a significant effect of creativity anxiety on the difference in anxiety ratings between the Analogy Finding Matrix task with and without the creativity cue (*p* > .05), suggesting that those who are higher and lower in creativity anxiety experience similar changes in state anxiety between the two tasks.

**Figure 3:**
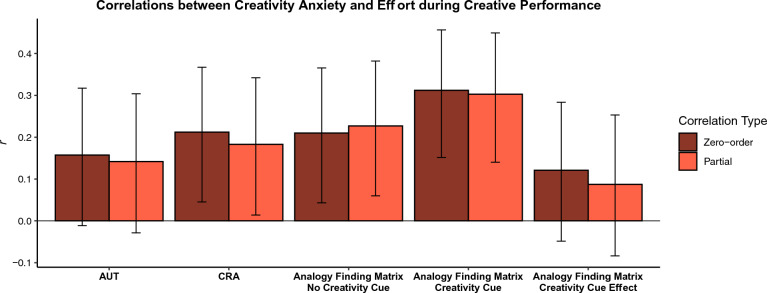
Shows associations between creativity anxiety and levels of effort participants reported exerting during creative performance. The darker red bars indicate zero-order Pearson’s correlations. The lighter red bars indicate partial correlations controlling for non-creativity anxiety control scores and general trait anxiety. Error bars represent 95% confidence intervals.

Next turning to effort, Fig. 3 shows both zero-order correlations between creativity anxiety and reported effort during each task and partial correlations controlling for non-creativity anxiety control scores and for general trait anxiety. Results indicate significant zero-order associations between creativity anxiety and all effort measures with the exception of effort during the AUT (all other *p*s < .05). Controlling for non-creativity anxiety control scores and general trait anxiety resulted in the effect on CRA effort becoming non-significant, however. The observed results suggest that creativity anxiety is associated with greater, not lesser, exerted effort during creativity tasks, though the effects on effort are not particularly consistent. While heterogeneous, these results appear more in line with the compensatory effort hypothesis (i.e., that those higher in creativity anxiety would exert additional effort on creativity tasks, the rationale for which comes from work on trait anxiety and effort^18^) as opposed to the effort avoidance hypothesis (i.e., that those higher in creativity anxiety would avoid exerting effort during creativity tasks, evidence for which comes from work on math anxiety and proxies for effort^22^). We do not observe a significant effect of creativity anxiety on the difference in effort ratings between the Analogy Finding Matrix task with and without the creativity cue (*p* > .05), suggesting that differential effort between the two tasks does not occur as a function of creativity anxiety.

**Figure 4:**
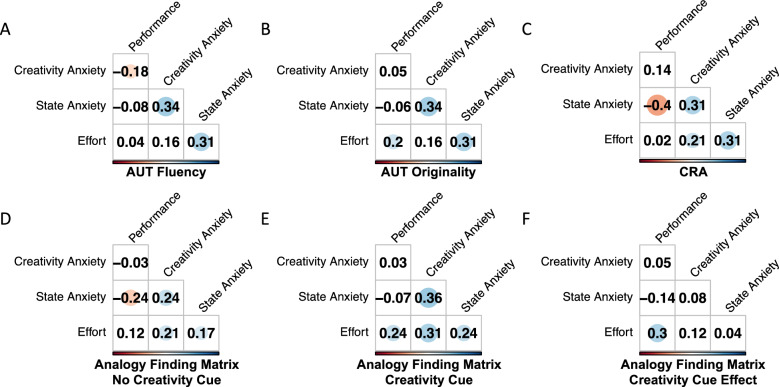
Shows zero-order Pearson’s correlations between creativity anxiety and performance, state anxiety, and effort associated with each creativity measure. The size and brightness of circles conveys the size of the association; associations that lack a circle are not significant.

The above analyses provide evidence that, for all of the tasks included in this work, creativity anxiety is predictive of state anxiety and/or effort participants reported during the task (though it was not predictive of differences in anxiety or effort between the uncued and creativity-cued Analogy Finding Matrix Task). Nevertheless, in Analysis 1 we found that creativity anxiety was only associated with some measures of creative performance. Next, we therefore planned to assess associations between both of the state-level measures we collected—anxiety and effort—and performance on each of the tasks. When considering these associations, we also wished to provide the full context of other interrelations among creativity anxiety and performance, state anxiety, and effort associated with each task. These interrelations are displayed in Fig. 4, in which the matrices indicate correlations between all of these variables for each task.

Results indicate inconsistent associations between measures of performance and state anxiety/effort associated with each task: state anxiety is significantly predictive of performance on the CRA and on the uncued Analogy Finding Matrix task, but not of any of the other performance variables. Effort, on the other hand, is predictive of AUT Originality and performance on the cued Analogy Finding Matrix task, but not the other tasks. We also found that the difference in effort participants expend between the uncued and cued Analogy Finding Matrix task is predictive of the difference in performance—the more people up their effort levels, the more they improve their performance.

An important goal of this study was to assess whether these state-level measures of anxiety and effort could explain any associations between creativity anxiety and creative performance. In terms of performance variables in this sample, we only observed significant associations between creativity anxiety and AUT Fluency. Neither state anxiety nor effort were predictive of AUT Fluency, however. As a result, even though creativity anxiety predicted heightened state anxiety associated with the AUT, state anxiety did not explain the association observed here between creativity anxiety and AUT Fluency—this association must therefore have occurred via alternate mechanisms.

To summarize findings from Analysis 2, we found that creativity anxiety was positively predictive of state anxiety and/or effort associated with each task. This result provides important evidence that creativity anxiety is, indeed, predictive of state-level factors associated with creative performance. We found inconsistent links, however, between these state-level factors and performance. This inconsistency may help to explain, in part, why creativity anxiety was weakly and varyingly predictive of creative performance—paths between creativity anxiety, state-level factors like anxiety and effort, and performance are complex. As a result, major mechanisms that we hypothesized would, in part, lead to associations between creativity anxiety and creative performance did not appear to operate in the simple explanatory ways we hypothesized in the context of the present paradigm. This may also help account for why the observed creativity anxiety-performance association we did find in this sample, that between creativity anxiety and AUT Fluency, was fairly weak: while there appears to be some other mechanism(s) giving rise to an association between creativity anxiety and the number of ideas people were able to generate, only nominal effects of state-level factors on performance were observed for this task.

## Discussion

Past work has provided evidence that creativity anxiety is associated with reduced levels of real-world creative achievement^3,4^, signifying that it may act as a barrier to the fulfillment of creative potential. Until now, however, whether creativity anxiety is associated with the ability to perform well on laboratory-based tasks measuring creative thinking, or with state levels of anxiety or effort during creative performance remained an open question. In the present work, we addressed this gap by testing whether creativity anxiety was predictive of multiple forms of creative cognitive performance. Results indicated that creativity anxiety was predictive of some forms of creative performance, such as AUT Fluency and the Analogy Finding Matrix task without a cue to be creative, but not other forms of creative performance, such as AUT Originality or the CRA, that we assessed here. Moreover, on tasks for which we *did* see significant associations between creativity anxiety and creative performance, the effect sizes of these associations were fairly small.

With regard to state levels of anxiety and effort during creative performance, we found that creativity anxiety was positively predictive of both anxiety and effort levels associated with creative tasks in most cases. However, while creativity anxiety generally predicted higher state anxiety and effort levels, these state-level factors themselves were only inconsistently associated with performance in the context of the present paradigm. This means that, in the context of the present work, those who were high in creativity anxiety often felt more anxious in-the-moment when having to think creatively, expended greater effort during creative performance, but ultimately performed about the same as their less anxious peers on many creativity tasks. Interestingly, on tasks in which there was a ‘correct’ answer—such as the CRA and the uncued Analogy Matrix task, state anxiety did seem to diminish performance. This effect, however, seemed to lessen or disappear when participants were asked to think creatively on the Analogy Matrix task, suggesting that permission to ‘think outside the box’ might be a simple method through which effects of state anxiety on creative performance might be mitigated. Broadly, the present findings suggest complex associations among creativity anxiety, state-level factors associated with thinking creatively, and the quality of creative performance. In particular, they raise questions about whether or in what contexts state anxiety and effort levels might have an impact on creative performance. Below, we discuss the implications of these findings, the limitations of the present work, and directions for future research on creativity anxiety.

One of the primary questions that arises from the results of this work concerns why creativity anxiety might be predictive of some of the metrics of creative performance we collected, but not others. For instance, we found that creativity anxiety predicted Fluency on the AUT (i.e., the number of different ideas participants were able to generate), but not Originality (i.e., how unique those ideas were). Moreover, we found that creativity anxiety predicted performance on the uncued Analogy Finding Matrix task (when participants were not explicitly cued to be creative), but it did not predict either performance on the same task when a creativity cue was present or the change in performance between these tasks. Finally, we found no evidence that creativity anxiety was predictive of CRA performance. Why might creativity anxiety be predictive of performance on some creative performance tasks but not others? An initial explanation could have hinged on the idea that those who are high in creativity anxiety only experienced heightened state anxiety on some of the tasks. Our state-level data, however, show that this is not the case—creativity anxiety was predictive of state anxiety for all the tasks we collected (with the exception of the Analogy Finding Matrix task when not cued to be creative, likely resulting in many participants not viewing this task as “creative”; see further discussion of this point below). This means that even on tasks like the CRA for which creativity anxiety was not predictive of performance, those who were higher in creativity anxiety still felt more anxious while completing the task.

One factor that could potentially explain why creativity anxiety predicted performance on some tasks but not others is the relevance of previous practice for task performance. In the math anxiety literature, one of the reasons why math anxiety is thought to be linked to poor math performance is that those who are math-anxious tend to avoid math when possible, thereby getting fewer opportunities to practice developing their math skills^19^. Forthcoming work provides some initial indications that creativity anxiety is similarly associated with a tendency to avoid pursuits that are perceived as involving creative thinking (Daker et al., under review). These findings suggest that it is likely that those who are high in creativity anxiety have a tendency to avoid situations that would enable them to develop their creative abilities. It is possible that the measures used in the present work differ in how dependent they are on previous experience with the type of creative thinking they test. The ability to fluently generate ideas, measured by AUT Fluency, may be especially practice-dependent, and if those who are anxious about thinking creatively avoid situations in which they will need to generate ideas, they may get less practice with this skill and subsequently develop it to a lesser extent than those who are less anxious about creative thinking. Other metrics of creative performance, like the originality of generated ideas, or performance on the CRA, may be less reliant on past experience participants are likely to have had. Another possibility in which the causal direction is reversed is that those who know they struggle to generate new ideas are more likely to develop higher levels of creativity anxiety—evidence for this phenomenon can be found in the analogous math anxiety literature, where early poor math performance is predictive of later development of higher math anxiety levels^43^. While future work would be needed to more fully understand precisely why creativity anxiety is associated with AUT Fluency, the present results suggest that further investigation into the link between fluent idea generation and creativity anxiety is worthwhile.

Considering the other performance measure of which creativity anxiety was predictive, a potentially illuminating finding from the present work was that, perhaps surprisingly, creativity anxiety was predictive of performance on the Analogy Finding Matrix task when there was *no* cue to be creative, but it was not predictive of performance on the same task when a cue to be creative was present. Why might this be? The most likely explanation, from our perspective, is that this discrepancy can be explained by the mindset with which participants are completing the uncued Analogy Finding Matrix task. To score highly on the Analogy Finding Matrix task, one must identify not only semantically proximate (less creative) analogies (e.g., “Kitten is to Cat as Puppy is to Dog”), but also multiple more semantically distant (creative) analogies (e.g., “Kitten is to Cat as Spark is to Fire”). The finding that creativity anxiety is predictive of performance on the Analogy Finding Task when there is no explicit indication that this task is a creativity task may reflect differences in how those who are high vs. low in creativity anxiety approach the task. Those who are high in creativity anxiety may approach this task with fairly low “state creativity”^41^—their mindset going into this may be to select only the analogies that are most straightforward rather than actively seeking creative possibilities when not instructed to do so. Those who are lower in creativity anxiety, however, may approach the task with higher state creativity, being inclined to actively seek out more creative combinations of ideas even when not explicitly prompted to do so. From this perspective, the uncued Analogy Finding Matrix task may provide an index of participants’ baseline state creativity, and this finding may suggest that when presented with open-ended situations, those who are higher in creativity anxiety are less likely to spontaneously think creatively than those who are lower in creativity anxiety. While those who are higher in creativity anxiety perform less creatively on this task when not explicitly prompted to be creative, a perhaps optimistic finding from this work is that when they *are* prompted to be creative, they are able to perform just as well as their less anxious peers. This pattern may suggest that, at least for some forms of creativity, those who are high in creativity anxiety possess the potential to perform well and that consciously adopting a creative mindset can help them reach their potential.

In addition to assessing associations between creativity anxiety and creative performance, a primary objective of this study was to investigate state-level dynamics of creative performance (state anxiety and effort) as a function of creativity anxiety. We found that high levels of creativity anxiety were associated with greater state anxiety for each task (with the exception of the Analogy Finding Matrix task when there was no cue to be creative after controlling for general trait anxiety and for non-creativity anxiety control scores, perhaps because participants did not perceive this task as “creative”), suggesting that those who are high in creativity anxiety do need to contend with higher state anxiety levels during creative performance. This evidence supports a key assumption in the creativity anxiety literature, namely that those who report higher levels of trait-level anxiety toward being creative will also report higher state-level anxiety associated with creative performance. Future work could follow up on this finding by assessing whether individual differences in emotion regulation processes moderate the link between trait-level creativity anxiety and state-level anxiety while engaging in creative thinking. It is possible that individuals that are high in creativity anxiety but that possess effective emotion regulation capacity can avoid feeling anxious in-the-moment when it is time to think creatively and that those with low emotion regulation capacity would feel especially anxious when faced with a need to be creative—we recently found that this was the case for those high in math anxiety^44^, and similar dynamics might be at play for creativity anxiety as well. When we considered effort, we found that, for many tasks, creativity anxiety was significantly positively associated with reported effort during creative performance. Additionally, even for the tasks for which the association between creativity anxiety and effort was not significant, the association was still in the positive direction. While the strength of the results are somewhat varying, this pattern suggests that those who are higher in creativity anxiety are more likely to be trying harder during creative performance. This is in line with findings from the general anxiety literature that those who are high in trait anxiety expend more effort on cognitive tasks^24^, but differs from evidence in the math anxiety literature that those who are math-anxious choose to expend less effort on math tasks^22,23,45^. Turning to implications for performance, interestingly, state anxiety and effort were only inconsistently associated with creative performance in the context of this study. While creativity anxiety was often predictive of these state-level factors, it was never the case that both creativity anxiety and a state-level factor predicted performance on a task, a prerequisite for testing whether these state-level factors mediated the link between creativity anxiety and performance. These results help characterize the experience of creative performance for participants who were higher in creativity anxiety—they typically felt more anxious and expended additional effort during performance, but ultimately often performed just as well as those who were lower in creativity anxiety. They also suggest that, at least for the tasks collected here, associations between creativity anxiety and creative performance likely occur as a result of mechanisms other than state anxiety and effort (including the possible mechanisms discussed above). Future work could seek to understand whether there are differential effects of state anxiety and effort on creative performance for individuals who perceive the stress induced by creativity anxiety as more of a challenge or a threat, as past work has found that this is a key individual difference that determines outcomes under stress^46–52^.

Importantly, while state-level anxiety and effort do not appear to consistently predict performance in the present work, the finding that creativity anxiety is associated with heightened state anxiety and effort levels during creative performance may still be consequential. People tend to avoid situations in which they will be anxious^53^ and situations in which they will have to expend high amounts of cognitive effort^54^. These in-the-moment feelings of anxiety and having to try harder may therefore play a contributing role in avoidance of real-world creative pursuits by those high in creativity anxiety. This may also lead to less persistence when people do take on creative pursuits: regularly experiencing heightened anxiety and effortfulness as a part of academic courses or even careers that involve a great deal of creative thinking may lead individuals higher in creativity anxiety to drop out of these pursuits, even if they might otherwise enjoy them. Finding ways to reduce the anxiety and effortfulness that those high in creativity anxiety experience when they have to be creative, if done over time, may therefore result in a lower tendency to avoid or desist from pursuits that involve creative thinking.

The heightened state-level anxiety and effort that those high in creativity anxiety experience may have other important consequences as well. In the math anxiety literature, there is evidence that for math anxiety to impact cognitive performance, the working memory demands of the task need to be sufficiently high. The reason why state anxiety is thought to negatively impact math performance is that this anxiety coopts limited working memory resources that are necessary for successful performance^20,55, 56^. If the working memory demands of a task are low, however, participants may have more than enough working memory resources to meet these demands even if anxiety is depleting some of these resources. Importantly, there continues to be disagreement over the extent of working memory demands associated with creative performance^57–59^. It is therefore possible that the working memory demands of many of the tasks we employed were not sufficiently high to reach a point where in-the-moment reductions in working memory capacity by state anxiety would interfere with performance. Relevant to this is that past meta-analytic work has found only small effects of state anxiety on creative performance^16^. Another possibility is that in this online study overall anxiety levels may have been suppressed compared to, say, having to participate in a study in a more formal lab setting where an experimenter is present (for evidence suggesting greater anxiety for in-person assessment compared to online assessment, see Pirrone et al.^60^). If this were the case, it could be that the anxiety levels observed in this study simply were not great enough to disrupt working memory resources sufficiently to lead to performance deficits.

It seems quite likely that higher state anxiety and effort associated with creativity anxiety would represent a disadvantage in contexts that involve more demanding tasks, more competing demands on working memory resources, additional sources of stress/anxiety, and/or the need to sustain performance over extended periods of time. Any of these conditions would deplete the working memory resources available for task performance, and, importantly, any (or all) of these conditions is frequently encountered in real-world contexts where creative thinking is required. Take, for instance, a work meeting where employees need to pitch new ideas to their boss. In such a situation, one would need to (A) generate ideas, (B) evaluate whether each one is worth sharing, (C) overcome interference from hearing the ideas of others, (D) deal with the potential anxiety that might be inherent to a situation that involves being evaluated by both peers and someone in a position of authority, and (E) sustain all of this consistently throughout the whole meeting, and on a day-to-day basis in the context of a job that involves creative thinking. In real-world situations like these, where creative performance needs to occur in the context of many other taxes on cognitive resources, creative performance may be particularly likely to be affected by heightened anxiety levels or effortfulness that occur as a result of high levels of creativity anxiety. Indeed, consistent with this idea, past work has shown that creativity anxiety is negatively predictive of real-world creative achievement^3,4^. Testing whether the state-level effects of creativity anxiety are more impactful on creative performance in demanding real-world situations, then, could be an important next step in better understanding other potential impacts of creativity anxiety on creative performance. Assessing links between creativity anxiety and real-world creative performance could be an especially worthwhile research direction, as poor real-world creative performance may, itself, lead to elevated creativity anxiety levels in the future (for evidence of underperformance leading to future heightened cognition-specific anxiety levels, see Gunderson et al.^43^).

The present work had several limitations. First, as discussed, this initial investigation was limited to only a few types of creative performance using lab-based tasks—associations between creativity anxiety and other forms of creative performance should be investigated in the future, particularly those that measure ability within more real-world creative domains like creative writing or visual art. Additionally, our AUT measure included only one item (brick). While this is fairly common in the literature, future work could include other AUT items to get a more expanded measure of divergent idea generation ability and its relation with creativity anxiety. Second, all of the data collection in this work took place online, and effect sizes are often smaller in online research^33^ than in-person data collection. We compensated for this with a large sample size, but it is possible that some of the effects we tested for require the additional feeling of being assessed that is provided by completing assessments in a lab as opposed to the comfort of one’s own home. For instance, state anxiety levels may have been higher if this work took place in a lab-based setting where assessment pressures were higher, and it is possible that this would have reached a threshold at which impacts of state anxiety on performance would have been more evident. Third, while we believe our measure of the effortfulness of task performance was informative, it is still possible some responding on this measure was driven by demand characteristics (i.e., participants not wanting to report low effort levels, which could have been interpreted as disqualifying to receive compensation for the study). We did not observe ceiling effects on any of these effort measures, but it is still possible that demand characteristics drove some responding.

## Conclusion

Recently, we have proposed that creativity anxiety, or anxiety toward creative thinking, can act as an affective barrier to the fulfillment of creative potential. Past work has found that creativity anxiety is predictive of measures of real-world creative achievement, providing initial evidence for this idea. In the present work, we extended this line of research by, for the first time, testing whether creativity anxiety is associated with poor creative cognitive performance on lab-based tasks. Results showed that creativity anxiety was weakly predictive of the number of unique ideas participants are able to generate (AUT Fluency) and of performance on a creative analogy task when the task was not explicitly framed as creative (Analogy Finding Matrix task without a creativity cue), but none of the other creative performance metrics we collected. This work therefore provides evidence that creativity anxiety is associated with creative performance, but, at least in the present context, not all forms of creative performance. However, creativity anxiety was found to generally be associated with increased state anxiety and effort reported during creative performance, suggesting that those higher in creativity anxiety may be more likely to underperform in situations where the creative tasks are more working memory-demanding or when there are external factors imposing additional cognitive load. It is our hope that this initial investigation into associations between creativity anxiety and creative performance will lay the groundwork for both future work that tests associations with other forms of creative performance, and also for intervention work to attempt to alleviate any negative effects of creativity anxiety on creative performance. Better understanding both any negative outcomes creativity anxiety predicts and, crucially, the mechanisms by which these associations are realized can allow us to most effectively help those who do not yet feel comfortable thinking creatively fulfill their creative potential.

## Data Availability

The data supporting this work can be found at: https://osf.io/ycxvn/?view_only=0ade9e081c8f4310b123eef2b4dfa7b8.
